# Heart Rate and Systolic Blood Pressure Variability in the Time Domain in Patients with Recent and Long-Standing Diabetes Mellitus

**DOI:** 10.1371/journal.pone.0148378

**Published:** 2016-02-05

**Authors:** Ana Leonor Rivera, Bruno Estañol, Horacio Sentíes-Madrid, Ruben Fossion, Juan C. Toledo-Roy, Joel Mendoza-Temis, Irving O. Morales, Emmanuel Landa, Adriana Robles-Cabrera, Rene Moreno, Alejandro Frank

**Affiliations:** 1 Centro de Ciencias de la Complejidad, Universidad Nacional Autónoma de México, México, D.F., México; 2 Instituto de Ciencias Nucleares, Universidad Nacional Autónoma de México, México, D.F., México; 3 Centro de Física Aplicada y Tecnología Avanzada, Universidad Nacional Autónoma de México, Querétaro, México; 4 Laboratorio Nacional de Ciencias de la Complejidad, México, D.F., México; 5 Laboratorio de Neurofisiología, Departamento de Neurología y Psiquiatría, Instituto Nacional de Ciencias Médicas y Nutrición Salvador Zubirán, México, D.F., México; Niigata University Graduate School of Medical and Dental Sciences, JAPAN

## Abstract

Diabetes Mellitus (DM) affects the cardiovascular response of patients. To study this effect, interbeat intervals (IBI) and beat-to-beat systolic blood pressure (SBP) variability of patients during supine, standing and controlled breathing tests were analyzed in the time domain. Simultaneous noninvasive measurements of IBI and SBP for 30 recently diagnosed and 15 long-standing DM patients were compared with the results for 30 rigorously screened healthy subjects (control). A statistically significant distinction between control and diabetic subjects was provided by the standard deviation and the higher moments of the distributions (skewness, and kurtosis) with respect to the median. To compare IBI and SBP for different populations, we define a parameter, α, that combines the variability of the heart rate and the blood pressure, as the ratio of the radius of the moments for IBI and the same radius for SBP. As diabetes evolves, α decreases, standard deviation of the IBI detrended signal diminishes (heart rate signal becomes more “rigid”), skewness with respect to the median approaches zero (signal fluctuations gain symmetry), and kurtosis increases (fluctuations concentrate around the median). Diabetes produces not only a rigid heart rate, but also increases symmetry and has leptokurtic distributions. SBP time series exhibit the most variable behavior for recently diagnosed DM with platykurtic distributions. Under controlled breathing, SBP has symmetric distributions for DM patients, while control subjects have non-zero skewness. This may be due to a progressive decrease of parasympathetic and sympathetic activity to the heart and blood vessels as diabetes evolves.

## Introduction

Cardiovascular dysfunction is a common Diabetes Mellitus (DM) complication due to autonomic neuropathy [1–5]. It has been traditionally diagnosed by its clinical manifestations such as postural hypotension, persistent tachycardia or fixed heart rate [6, 7]. More recently, DM has been associated with alterations in the dynamics of the systolic blood pressure (SBP), and with a decreased heart rate variability (HRV) [2, 3, 8]. Parasympathetic tone decreases heart rate and cardiac contractility, whereas activity of the sympathetic branch opposes these effects at the heart level and also regulates peripheral vasoconstriction [9]. Loss of this balance (which is regularly the case in DM patients) is associated with a high risk of cardiovascular disease [10, 11]. Under resting conditions, vagal tone prevails and variations in the heart period are largely dependent on vagal modulation [12]. Vagal and sympathetic modulation of the sinus node can be evaluated through HRV providing a non-invasive method for understanding cardiac autonomic control [13–15]. HRV has a temporal structure with robust long-range correlations, fractal and non-linear features, which have been found to break down under pathologic conditions like DM, reflecting changes in the neuro-autonomic control mechanisms [3, 16, 17].

The quantification of cardiovascular function in DM usually is performed through the standard cardiovascular reflex tests established by the American Diabetes Association [2, 18]: supine rest recording; heart rate measurement during deep timed breathing; the Valsalva maneuver and standing up, to assess cardiac parasympathetic and sympathetic cardiac activity and blood pressure responses; and sustained handgrip to evaluate sympathetic nervous activity to the heart and blood vessels (so-called central command). However, several of these conventional tests have low correlations [18, 19]. Therefore, we decided to explore methods like HRV and SBP variability in the time domain, in particular higher statistical moments like kurtosis and skewness with respect to the median, which are not used often to differentiate vagal and sympathetic modulation of the autonomic nervous system. We also introduce a new parameter α, defined as the ratio of the radius of the moments for interbeat interval IBI (standard deviation, skewness and kurtosis with respect to the median) and the same radius for SBP. As far as we could check, this is the first time that the effects of heart rate variability and blood pressure variability are evaluated in a single parameter. Short-term and/or long-term HRV have been utilized to study DM [6, 18–24]. Reduction of time–domain parameters of HRV, especially of standard deviation, has been used as an early sign of DM autonomic neuropathy [20, 21]. DM has been usually characterized in 24 hours Holter HRV records and in simple bedside tests [22], but there are few conclusive studies using short-term registers such as the 5 minute records analyzed here. In large study meta-analyses, DM microvascular complications have been correlated in visit-to-visit SBP variability [25–28], but the research articles that consider beat-to-bead blood pressure variability in DM patients are few [29, 30]. It has been reported that DM patients can be differentiated from healthy subjects because they show less variability in diastolic blood pressure (DBP) during deep breathing, failure to exhibit DBP decreases during recovery from a cold pressure stimulus, a flatter DBP response pattern when changing from sitting to standing [31], and abnormal baroreceptor-cardiac reflex sensitivity (BRS) possibly due to abnormal parasympathetic function [32]. Moreover, SBP has smaller changes in detrended fluctuation analyses during active standing and handgrip, and measures of the IBI reflect lower parasympathetic cardiac activity at rest [33]. Interestingly, whereas heart rate variability in rest appears to be a protective health factor [6], it has been suggested that blood pressure variability is a risk factor [34–37].

The objective of this study is to evaluate the influence of DM type II disease on the heart rate and blood pressure variability. Hence, we investigated how cardiac dynamics changes in patients with DM type II by analyzing in the time domain the characteristics of IBI and SBP records simultaneously taken from subjects during clinostatism, orthostatism and controlled breathing tests. As far as we know, this is the first global analysis involving simultaneous IBI and SBP variability data using measures in the time domain to compare control subjects, recently diagnosed and long-standing DM patients.

## Research Design and Methods

All subjects provided written informed consent, underwent a screening history and physical examination. The patients with diabetes were part of a cohort of patients with metabolic syndrome who underwent a yearly glucose tolerance test. The Ethics’ Committee of the Instituto Nacional de Ciencias Médicas y Nutrición “Salvador Zubirán” approved the protocol for data recording. DM patients had a medical diagnosis by an expert, with blood glucose levels larger than 200 mg/dL at two hours in a glucose tolerance test, in agreement with the “Asociación Mexicana de Diabetes” and the 2011 “American Diabetes Association” recommendations [6].

This study did not consider the effects of age and gender because HRV of healthy subjects only shows small differences in standard deviation between young and old subjects and are independent of gender [18]. Body mass index (BMI) apparently also did not affect heart rate and blood pressure variability. Nevertheless, a recent study shows small changes in HRV due to sex and physical activity in the age group of 12–17 years [38]. We do not have subjects in that age range, and all our patients have similar physical activity patterns, and since scaling and non-linear properties of HRV and SBP remain on average unaffected by sex, age and BMI, we attributed the observed changes in our study to the autonomic regulation associated with DM.

Data were taken with a Portapres^®^ device (The Netherlands) to record simultaneously the blood pressure and the heart rate from:

- 30 healthy control subjects, 17 female, 13 male, with a BMI from 19.1 to 28.5 kg/m^2^ (with a mean and standard deviation of 24±3 kg/m^2^), and from 21 to 50 years old (33±8 yr). Subjects were classified as healthy if they did not smoke, had no cardiac diseases, and did not take medication. They were not hypertensive and had blood pressure levels of 120/80 mmHg or less.
- 30 patients with recently diagnosed DM type II, 16 female, 14 male, with BMI from 25.8 to 29.4 kg/m^2^ (27±2 kg/m^2^), and between 38 and 48 years old (41±5 years). These recent DM patients have been screened regularly every year with an oral glucose tolerance test of 75 g for two hours. In their previous medical examination (done at most 2 years before the diagnosis), they were considered to have metabolic syndrome but did not take medications.
- 15 patients with long-standing DM type II diagnosed 15±9 years previous to the present study, 10 female, 5 male, with BMI from 18.1 to 36.8 kg/m^2^ (27±6 kg/m^2^), and between 20 and 76 years of age (53±18 yr).

Control subjects and DM patients abstained from caffeine, beta-blockers, anticholinergics, antihistamines, opioids and adrenergic medication for the 48 hours before the test. IBI and SBP were registered simultaneously while the subject was in supine position (clinostatic record) for 5 minutes. Subjects were made to stand up, relaxed for 1 minute and stayed in this position for a 5 minutes standing registration (orthostatic record). Finally, a controlled breathing test at 0.1 Hz was done (subject was asked to inspire and expire maximally at six breaths per minute, 5 seconds in and 5 seconds out when it was standing up). Data consisted of short-term 5 minutes recordings measured non-invasively with the Portapres^®^ equipment.

**Fig 1:**
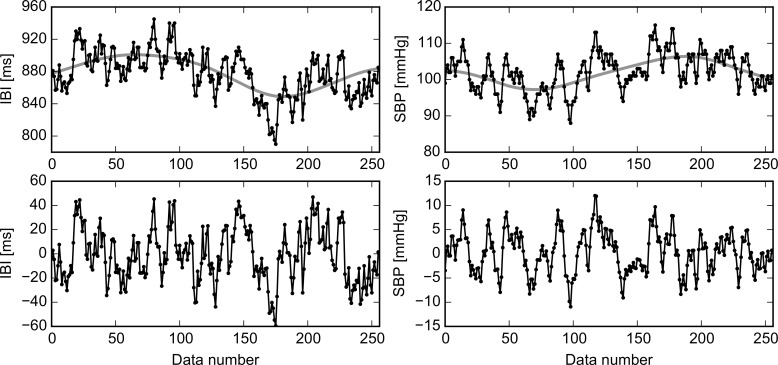
Signal detrending. Detrending of a typical IBI record (left-hand panels) and a SBP record (right-hand panels) for the same patient during the same maneuver. Original data points and the line of global trend are plotted in upper panels, while the detrended fluctuations appear in bottom panels.

Signals were detrended to avoid artificial contributions and to take out long oscillations with periods larger than the recording time of 5 min, i.e., with frequencies smaller than 0.04 Hz, known as very long frequency (VLF). In the literature it is recognized that VLF oscillations are due to regulatory mechanisms such as thermoregulation, while the autonomous nervous system is responsible of the heart rate oscillations from 0.04 to 0.4 Hz [3,12]. In Fig 1, original signals for IBI and SBP are shown, together with the global trend and the detrended fluctuations. Detrending was performed using the Empirical Mode Decomposition (EMD) technique [39]. To this end, a C implementation of the EMD algorithm was applied successively to extract higher-frequency modes (or IMFs) from the data until the remainder had few local extremes; this remainder was then considered as the global trend (see upper panels of Fig 1). This global trend was subtracted from the original data to deal only with the signal’s fluctuations (lower panels of Fig 1).

Traditionally, in the time domain, HRV is measured by the standard deviation (SD) of the interbeat intervals (IBI) [13], i.e. the square root of variance, which is the second moment with respect to the mean:

$$\text{SD}=\;\sqrt{\frac{1}{N-1}\sum_{i=1}^{N}{\left(x_{i}-\bar{x}\right)}^{2}},$$

where *N* is the number of data points recorded, summation is over all data points *x*_*i*_, and $\bar{x}$ is the population average (summation of all data points divided by *N*). Since variance is equal to the total power of spectral analysis, it reflects all the cyclic components responsible for variability in the recording period [13].

In the temporal domain, histograms of IBI for control subjects have a marked asymmetry toward the right hand side of the distribution [40, 41]. It has been proposed that asymmetric tails to the left or to the right reflect, respectively, the acceleration or deceleration capacity of the heart rate as an approximate distinction of vagal and sympathetic effects on the cardiac modulations [22]. Deviation from Gaussian symmetry can be measured by the skewness (the third moment of the distribution divided by the third power of SD) [42]. Traditionally, skewness evaluates deviations from the mean value, which is not an accurate measure of the center of asymmetric distributions, and does not allow to adequately separate HRV accelerations (deviations less than the central IBI, forming the left-hand portion of the histogram) from decelerations (greater deviations forming the right-hand part of the histogram). Usually, distributions of cardiovascular variables have a non-Gaussian distribution, so we reasoned it was more accurate to calculate the skewness, *sk*, as the third moment with respect to the median instead of the mean:

$$sk=\frac{1}{{\text{SD}}^{3}}\sum_{i=1}^{N}{\left(x_{i}-m\right)}^{3},$$

where *m* is the median, a quantity not as strongly affected by outliers or large deviations in the data, which is defined as the central value after the observations have been sorted in increasing order.

Another useful measure of the distribution, not widely used to analyze IBI and SBP time series, is the kurtosis, κ (the fourth moment) [42], a measure of how concentrated the data is around the mean:

$$\kappa =\frac{1}{{\text{SD}}^{4}}\sum_{i=1}^{N}{\left(x_{i}-m\right)}^{4}-3\;.$$

For a Gaussian (normal distribution), the kurtosis is zero, while positive κ corresponds to a leptokurtic distribution (more peaked than a Gaussian) and negative κ describes a platykurtic one (flatter than a Gaussian).

Here, graphs and statistical parameter calculations were done mainly using OriginPro^®^ 2015, 64 bits and independently checked using a code written in Python^®^ 2.7.7. We evaluated the first four moments for the IBI and SBP time series distributions. The IBI median is inversely proportional to the heart rate. Here we are interested only in the fluctuations of the heart rate, evaluated after the IBI time series is detrended. The standard deviation of the detrended IBI time series is one of the HRV measures of vagal and sympathetic modulation; skewness with respect to the median as a measure of symmetry reflects the balance of vagal and sympathetic effects, while the kurtosis measures the concentration of the data around the median and reflects the rigidity of the heart rate. Blood pressure variability is also analyzed through the SBP detrended time series, where also standard deviation, skewness with respect to the median and kurtosis are evaluated.

In order to compare the simultaneous IBI and SBP records, we define an α parameter as the ratio between the $\sqrt{{\left(\frac{SD}{m}\right)}^{2}+sk^{2}+\kappa^{2}}$ of the IBI and SBP records:

$$\alpha =\frac{{\left[\sqrt{{\left(\frac{\text{SD}}{m}\right)}^{2}+{\mathrm{sk}}^{2}+\kappa^{2}}\right]}_{\text{IBI}}}{{\left[\sqrt{{\left(\frac{\text{SD}}{m}\right)}^{2}+{\mathrm{sk}}^{2}+\kappa^{2}}\right]}_{\text{SBP}}}.$$

The definition of the α parameter can be explained as follows. A high heart rate variability (IBI) is considered being a protective factor for health and a low heart rate variability as a risk factor, whereas for blood pressure variability (SBP) the opposite appears to be true; therefore, we propose that in a single α parameter both heart rate and blood pressure risk factors can be quantified. Furthermore, as discussed above, successive moments of SD, *sk* and κ are required to describe fine details of variability of a signal and the concept of $\sqrt{{\left(\frac{SD}{m}\right)}^{2}+sk^{2}+\kappa^{2}}$, which could be interpreted as a sort of “distance” or “radius” in SD-*sk*-κ space. This is a useful way of synthesizing the various moments in a single measure in which the squaring of the quantities amplifies differences. Finally, SD has units whereas *sk* and κ are without units; therefore, we use the adimensional quantity SD/*m* (standard deviation normalized by the median) in order to be able to combined it with *sk* and κ in a single formula. This also allows to compare individuals with distinct heart rate baseline due to different SD on IBI.

Even when the histograms for each subject have tails and do not follow Gaussian distributions, the distributions of the moments for each group of study (30 control subjects, 30 recently diagnosed and 15 long-standing DM patients) are Gaussians (verified by Origin^®^ normality test). Student’s *t* test was used to compare the different study groups of control subjects, recently diagnosed DM patients, and long-standing DM patients. A value of *p*<0.05 was considered statistically significant in the hypothesis test of different means of the moments (standard deviation, skewness and kurtosis with respect to the median) and the α parameter.

## Results

**Fig 2:**
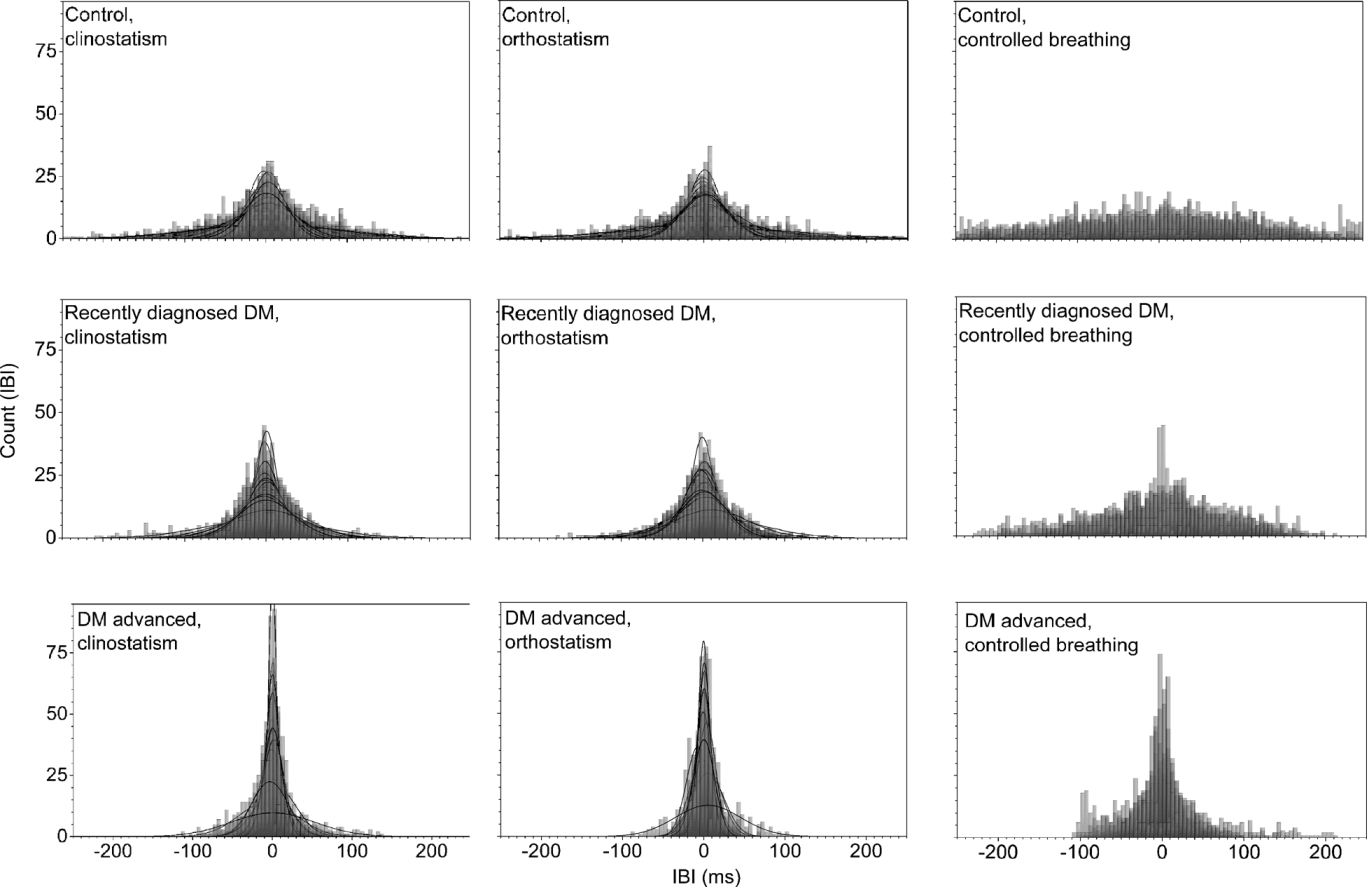
Histograms of detrended IBI time series for all subjects during clinostatism (left-hand panels), orthostatism (middle panels), and controlled breathing (right-hand panels). Superposed histograms are shown for all control subjects (upper row), recently diagnosed DM patients (middle row), and long-standing DM patients (bottom row).

**Fig 3:**
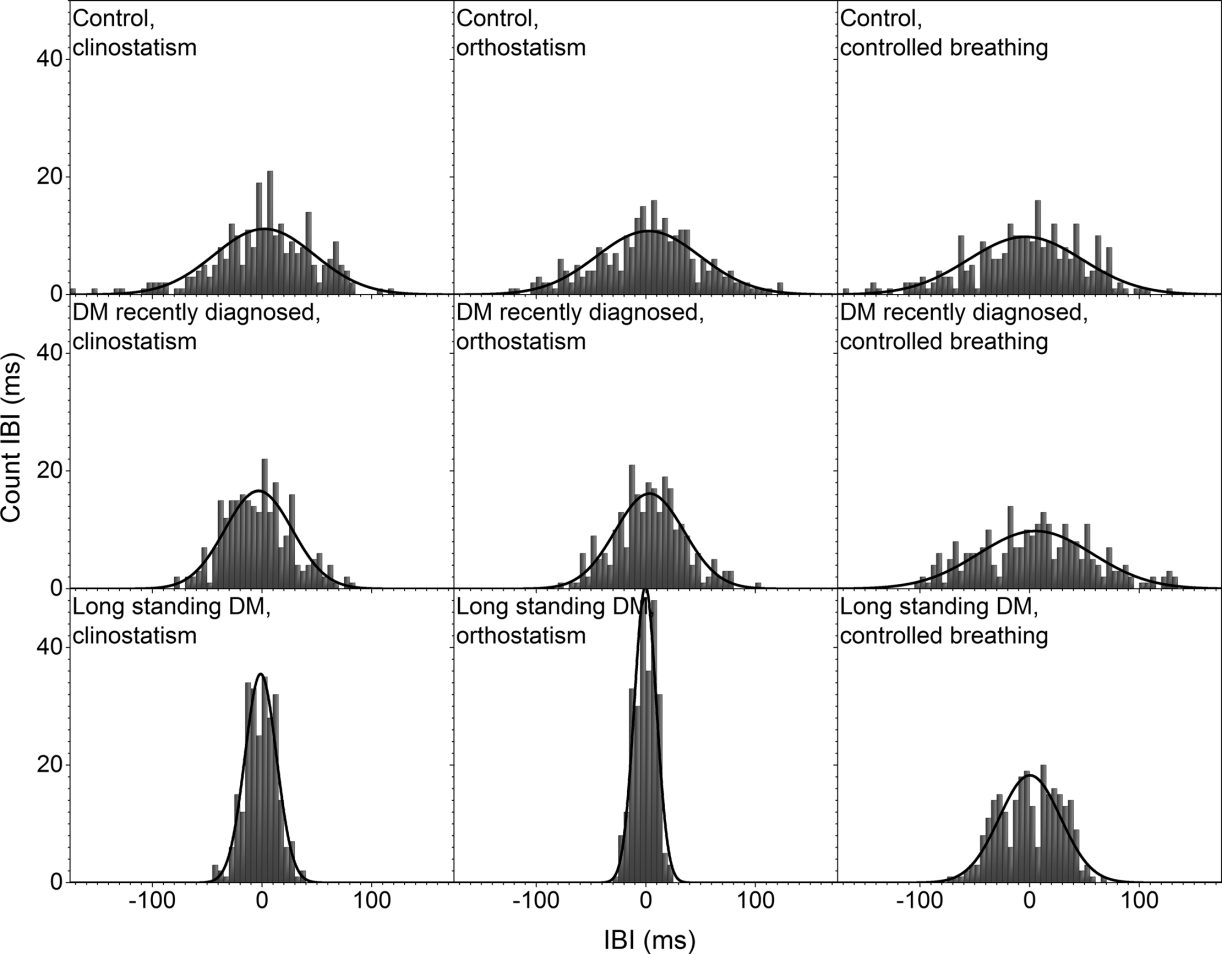
Histograms of detrended IBI time series for a typical control subject (upper row), a recently diagnosed DM patient (middle row), and a long-standing DM patient (bottom row), during clinostatism (left-hand panels), orthostatism (middle panels), and controlled breathing (right-hand panels).

Histograms of the detrended IBI time series for control subjects and DM patients during clinostatic, orthostatisc, and controlled breathing test are plotted in Fig 2, while Fig 3 shows the histograms of a typical control, recently diagnosed and long-standing DM patients under the different maneuvers. Continuous curves correspond to the best normal distribution fit to the histograms. From these figures, it is evident that as DM evolves the heart rate loses variability. In supine and standing positions, histograms of IBI time series of DM patients are more peaked than for control subjects, giving evidence that the heart rate dynamics becomes more rigid in disease, with smaller fluctuations and thus higher intensity and lower variability. Histograms of IBI signals during controlled breathing tend to be more platykurtic than during orthostatism and clinostatism.

**Fig 4:**
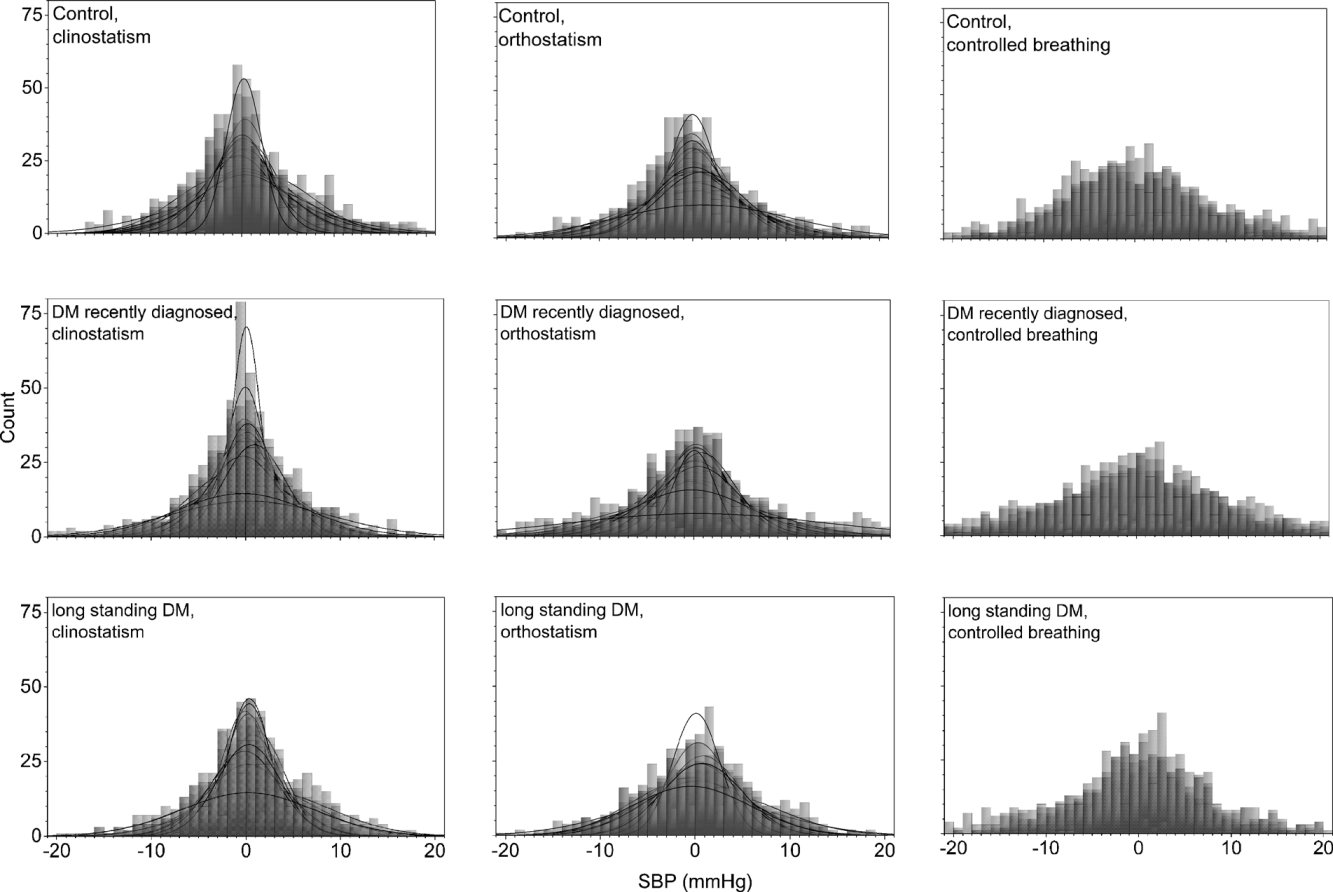
Histograms of detrended SBP time series for all the subjects during clinostatism (left-hand panels), orthostatism (middle panels), and controlled breathing (right-hand panels). Superposed histograms are shown for all control subjects (upper row), recently diagnosed DM patients (middle row), and long-standing DM patients (bottom row).

**Fig 5:**
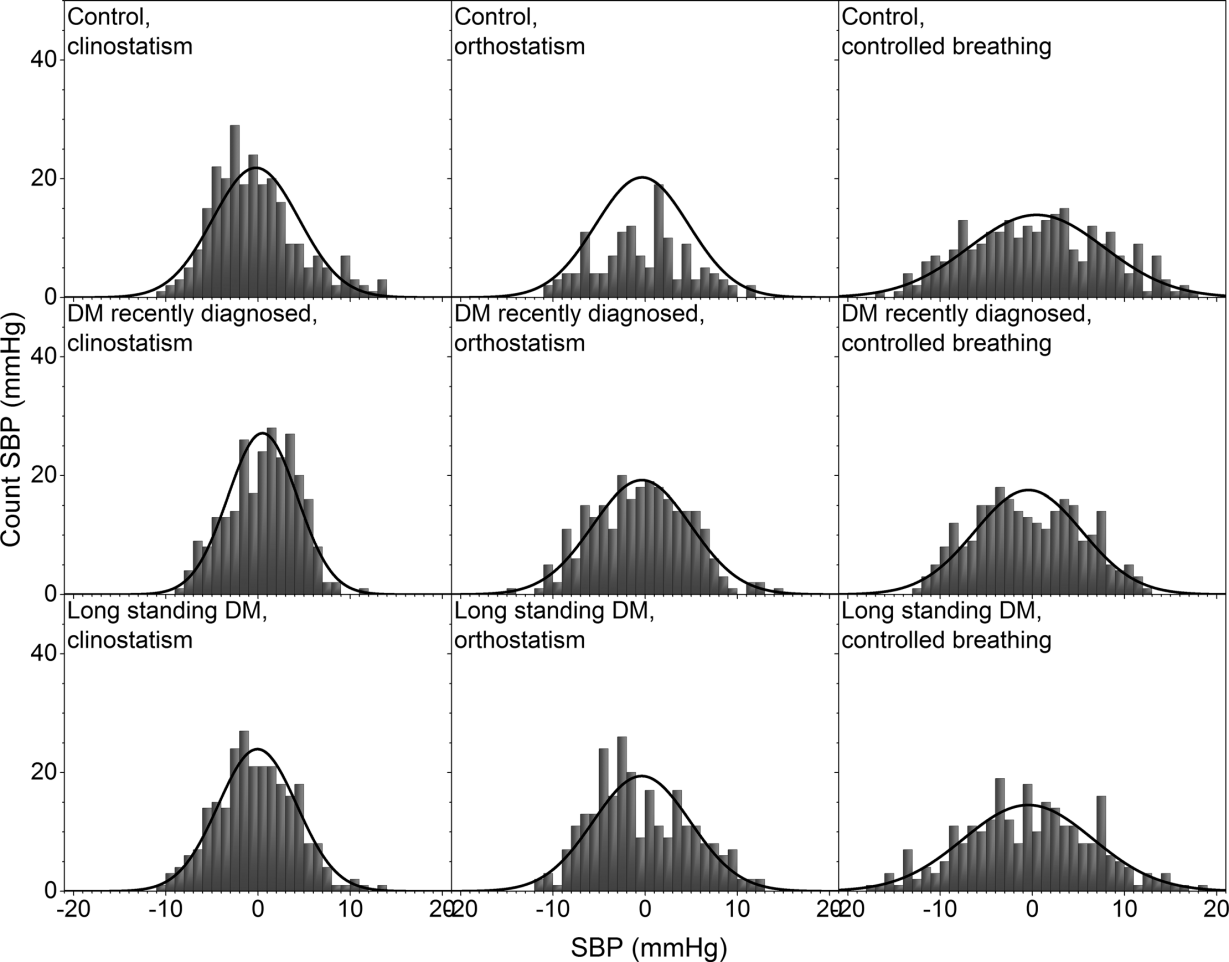
Histograms of detrended SBP time series for a typical control subject (upper row), a recently diagnosed DM patient (middle row), and a long-standing DM patient (bottom row), during clinostatism (left-hand panels), orthostatism (middle panels), and controlled breathing (right-hand panels).

Histograms of all the detrended SBP time series for control subjects and DM patients during clinostatic, orthostatisc, and controlled breathing test are plotted in Fig 4, while Fig 5 shows the histograms of a typical control subject, a typical recently diagnosed DM patient, and a long-standing DM patient during the different maneuvers. Continuous curves correspond to the best normal distribution fit to the histograms. From these figures, it is evident that the blood pressure shows less variability than the heart rate, and while IBI’s histograms are more peaked as DM evolves, SBP’s histograms are more peaked for control subjects than for long-standing DM patients. A high heart rate variability (IBI) is considered being a protective factor for health and a low heart rate variability as a risk factor, whereas for blood pressure variability (SBP) the opposite appears to be true. Therefore, we propose that both heart rate and blood pressure risk factors should be quantified in the α parameter. In supine position, histograms of SBP records of recently DM patients are more peaked than the ones for control subjects giving evidence for more rigid dynamics, while the histograms of long-standing DM patients look more similar to those of the control subjects. Moreover, during the clinostatism maneuver, SBP histograms have higher symmetry, and are leptokurtic in comparison with histograms in orthostatic condition. As DM evolves, blood pressure loses variability during orthostatism maneuver, thus, SBP histograms for DM patients give evidence for more rigid signals than the ones for control subjects. SBP histograms during controlled breathing are more platykurtic than the corresponding ones of orthostatism and clinostatism. However, blood pressure variability appears to be similar for control subjects and recently diagnosed DM patients while long-standing DM patients during controlled breathing histograms have smaller standard deviations, are more concentrated around the median, and more symmetric.

In supine position, the heart rate is influenced only by spontaneous breathing as gravity does not affect the blood-volume distribution in the body. IBI response is characterized by an asymmetric distribution with a tail to the right (see Figs 2 and 3) reflecting a preference for long IBI intervals and slower heart rate, i.e. sympathetic input stays constant and parasympathetic nervous system is activated (expiration) and deactivated (inspiration). IBI response when standing (orthostatic test), that reflects heart rate influenced both by breathing and gravity, approaches to a Gaussian distribution, becoming more symmetric showing a shift in the balance between sympathetic and parasympathetic nervous system with more input from the sympathetic nervous system to counter gravity by vasoconstriction in the lower extremities.

During controlled breathing, HRV mainly reveals the action of the hemodynamic of the lungs and heart, and the successive activation of vagal and sympathetic influences. In this case, the IBI histogram has a platykurtic distribution with a long tail to the right, associated again with the balance of vagus and sympathetic signals. In general, as diabetes evolves, HRV is more altered during the standing position. SBP histograms are more disperse and platykurtic than IBI ones, in fact, under orthostatism the SBP distribution is flatter over a wider range (compare Figs 2 and 4).

From a typical long-standing DM patient, the IBI record does not show a strong difference under clinostatism, orthostatism or controlled breathing (see Fig 3) indicating a loss of adaptation capacity to different stressors. Histograms have small variability, are symmetric and have leptokurtic distributions, reflecting a rigid heart response. Blood pressure dynamics is similar for all conditions (see Fig 5). SBP histograms are more disperse and platykurtic than IBI histograms (compare Figs 2 and 4).

**Fig 6:**
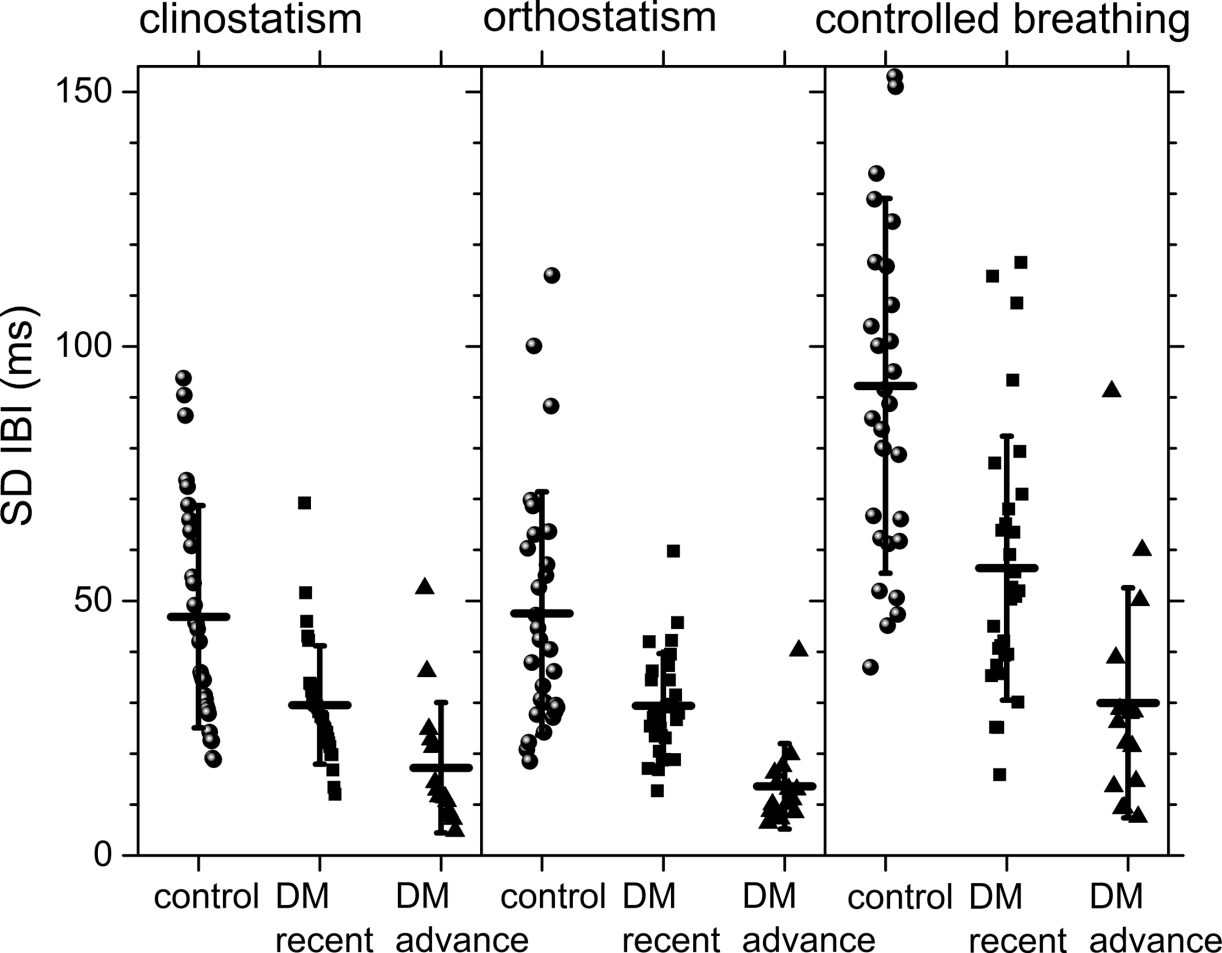
Standard deviation of detrended IBI data for the different groups during clinostatism (left-hand panels), orthostatism (middle panels), and controlled breathing (right-hand panels). The vertical crosshair corresponds to one standard deviation around the population average, while the horizontal one is the median.

The usual parameter employed to distinguish health from illness is the standard deviation of the IBI records, which we plot for comparison in Fig 6. This figure shows better separation of the groups in the clinostatism test but there is still a general overlap between groups.

**Fig 7:**
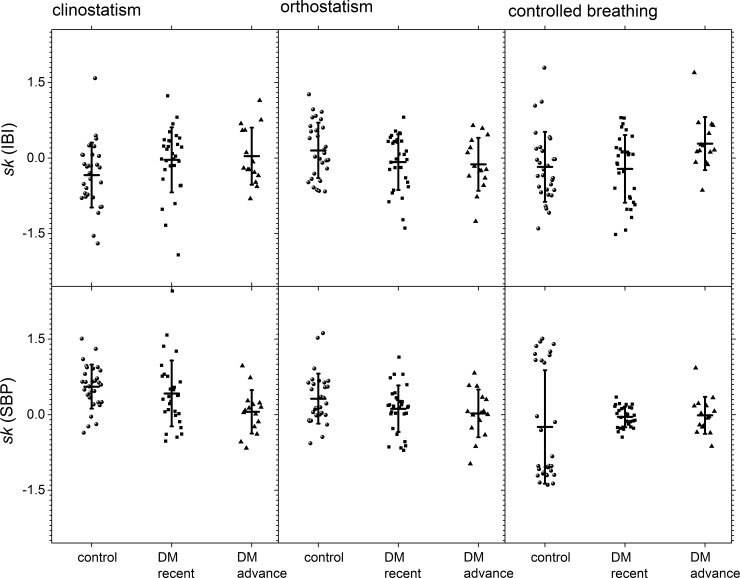
Skewness with respect to the median for the different groups during clinostatism (left-hand panels), orthostatism (middle panels), and controlled breathing (right-hand panels), IBI (top row), and SBP (bottom row). The vertical crosshair corresponds to one standard deviation around the population average, while the horizontal one is the median.

**Fig 8:**
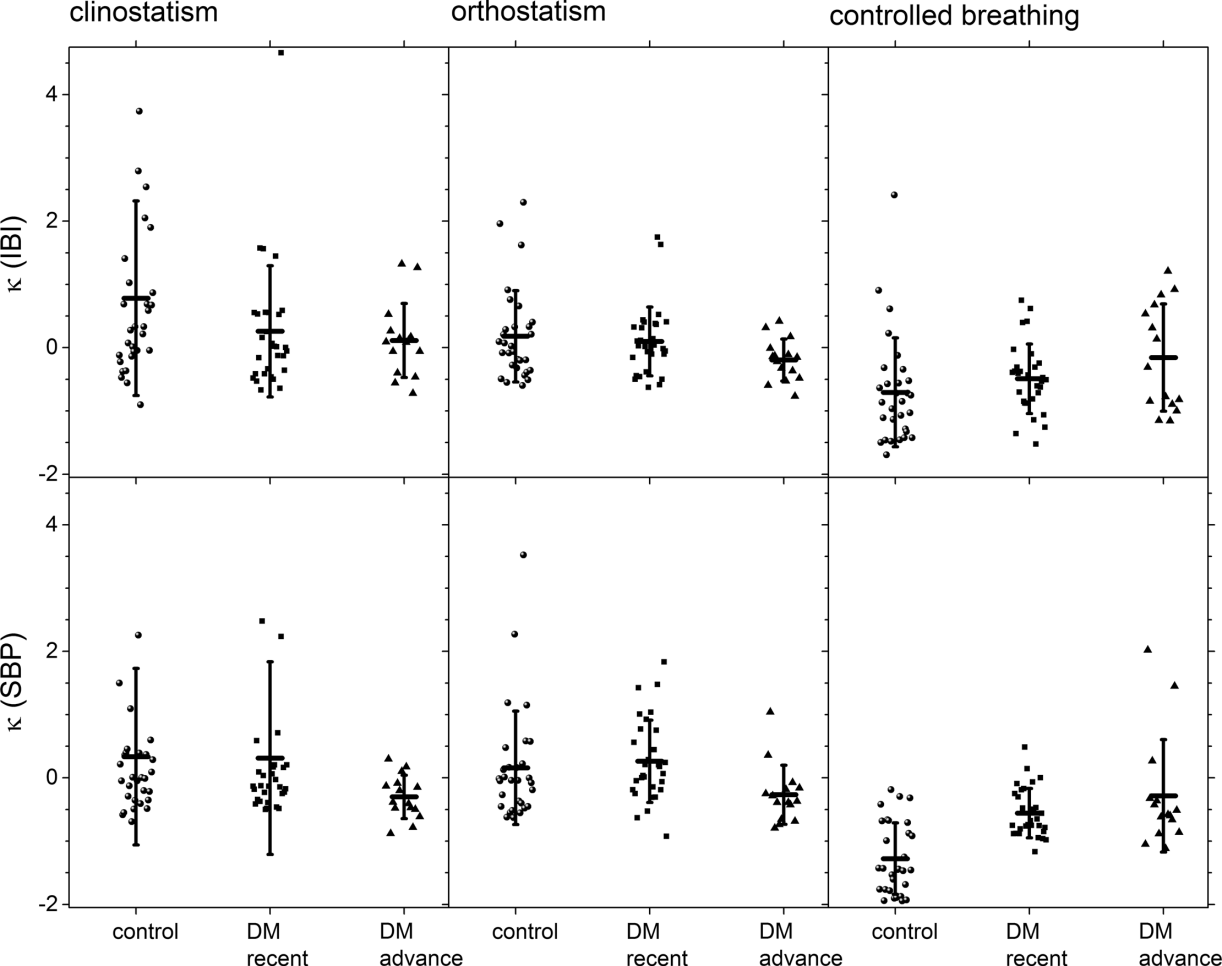
Kurtosis with respect to the median for the different groups during clinostatism (left-hand panels), orthostatism (middle panels), and controlled breathing (right-hand panels), IBI (top row) and SBP (bottom row). The vertical crosshair corresponds to one standard deviation around the population average, while the horizontal one is the median.

Moments of the distribution are shown in Fig 7 (skewness with respect to the median) and in Fig 8 (kurtosis with respect to the median) for control subjects, recently diagnosed DM patients and long-standing DM patients groups during all the maneuvers. In these graphs, it is possible to visually separate the different groups, especially for IBI during the clinostatic test and SBP under controlled breathing. In all these tests, control subjects present a greater data dispersion in IBI and SBP records than DM patients.

The statistical moments (cited hereafter as mean value ± SD) under all tests for heart rate variability are summarized in Table 1. Qualitatively, a negative skewness indicates that the tail on the left side of the probability density function is longer than the right side and the bulk of the values lie to the right of the mean, while a positive skewness indicates that the bulk of the values lie to the left of the mean [42]. In supine position, IBI’s skewness population average (Fig 7) for control subjects is negative (left-skewed distributions) and zero (symmetric) for recently diagnosed and long-standing DM patients, while IBI’s kurtosis population average (Fig 8) goes gradually from positive (leptokurtic distributions) for control subjects to zero (Gaussian) for long-standing DM patients. For the case of IBI under clinostatism (see Table 1), the t-student’s test for the hypothesis of distinct population average moments (SD, *sk*, *κ*) proved a statistically significant difference between control and long-standing DM patients, although there is an overlap due to the large variability in the statistics between subjects. Thus, the clinostatism test indicates that as DM evolves a more rigid cardiovascular response is produced, and IBI´s time series become more symmetric due to a loss of balance between vagal and sympathetic influence on the heart rate. The majority of the IBI values are on the right hand side with respect to the median reflecting a predominance of the vagal influence. In the standing up test only IBI’s kurtosis population average changes (Fig 8) from positive (leptokurtic distributions) for control subjects to negative (platykurtic) for long-standing DM patients crossing zero (Gaussian) for recently diagnosed DM patients. Finally, the controlled breathing test produces changes on IBI’s skewness population average (Fig 7) from zero (symmetric distribution) for control subjects to positive (right-skewed distribution) for long-standing DM patients. It is also noticeable that the time series of the control groups have the highest scatter.

**Table 1:** IBI’s statistical moments.

Maneuver	Statistical moment	Clinostatism	Orthostatism	Controlled breathing
Control	*m* (ms)	3.9 ± 7.4	1.2 ± 7.8	9.2 ± 23
	SD (ms)	47 ± 22	47 ± 23	92 ± 37
	*sk*	-0.2 ± 0.5	0.09 ± 0.33	-0.006 ± 0.37
	*κ*	0.8 ± 1.5	0.2 ± 0.7	-0.9 ± 0.7
Recently diagnosed DM II	*m* (ms)	0.7 ± 4.5	2.9 ± 7.4	8 ± 15
	SD (ms)	30 ± 12	29 ± 10	56 ± 26
	*sk*	-0.002 ± 0.5	-0.03 ± 0.37	-0.04 ± 0.33
	*κ*	0.2 ± 0.9	0.07 ± 0.48	-0.6 ± 0.6
Long- standing DM II	*m* (ms)	-0.8 ± 3.8	0.6 ± 3.6	-3.7 ± 12
	SD (ms)	17 ± 13	14 ± 8	30 ± 23
	*sk*	0.02 ± 0.37	-0.06 ± 0.28	0.2 ± 0.3
	*κ*	0.07 ± 0.56	-0.2 ± 0.3	-0.2 ± 0.9

median *m*, standard deviation SD, skewness *sk*, and kurtosis *κ*.

Statistical moments under all tests for blood pressure variability are summarized in Table 2. For the case of SBP (see Table 2), the t-student’s test for the hypothesis of distinct population average moments *sk* proved a statistically significant difference between control, recently diagnosed and long-standing DM patients; under clinostatism and orthostatism *κ* population average is statistically significant different for control and long-standing DM subjects. There is no difference on SBP’s SD and on *κ* under controlled breathing test. For all tests, SBP’s skewness population average (Fig 7) goes from positive (right-skewed distributions for control subjects) to zero (symmetric for long-standing DM patients) with a change of one order of magnitude. The most evident change in all the records analyzed is that for controlled breathing DM patients have symmetric distributions while control subjects have non-zero skewness, i.e., tails to the right or to the left (Fig 7). Under clinostatism and orthostatism, SBP’s kurtosis population average (Fig 8) goes from positive (leptokurtic distributions) for control subjects and recently diagnosed DM patients to negative (platykurtic) for long-standing DM patients.

**Table 2:** SBP’s statistical moments.

Maneuver	Statistical moment	Clinostatism	Orthostatism	Controlled breathing
Control	*m* (mmHg)	-0.26 ± 0.65	-0.04 ± 0.42	0.54 ± 0.94
	SD (mmHg)	4 ± 1	5 ± 2	6 ± 2
	*sk*	0.4 ± 0.3	0.2 ± 0.3	0.2 ± 0.3
	*κ*	0.3 ± 1.4	0.1 ± 0.8	-0.4 ± 0.4
Recently diagnosed DM II	*m* (mmHg)	-0.13 ± 0.68	0.15 ± 0.54	0.33 ± 0.84
	SD (mmHg)	4 ± 1	5 ± 2	7 ± 2
	*sk*	0.25 ± 0.45	0.09 ± 0.31	-0.04 ± 0.21
	*κ*	0.2 ± 1.4	0.3 ± 0.7	-0.5 ± 0.4
Long-standing DM II	*m* (mmHg)	0.45 ± 0.87	0.18 ± 0.89	0.2 ± 1.6
	SD (mmHg)	4 ± 2	5 ± 1	6 ± 2
	*sk*	0.05 ± 0.23	0.008 ± 0.255	-0.01 ± 0.37
	*κ*	-0.3 ± 0.3	-0.3 ± 0.4	-0.2 ± 0.9

median *m*, standard deviation SD, skewness *sk*, and kurtosis *κ*.

**Fig 9:**
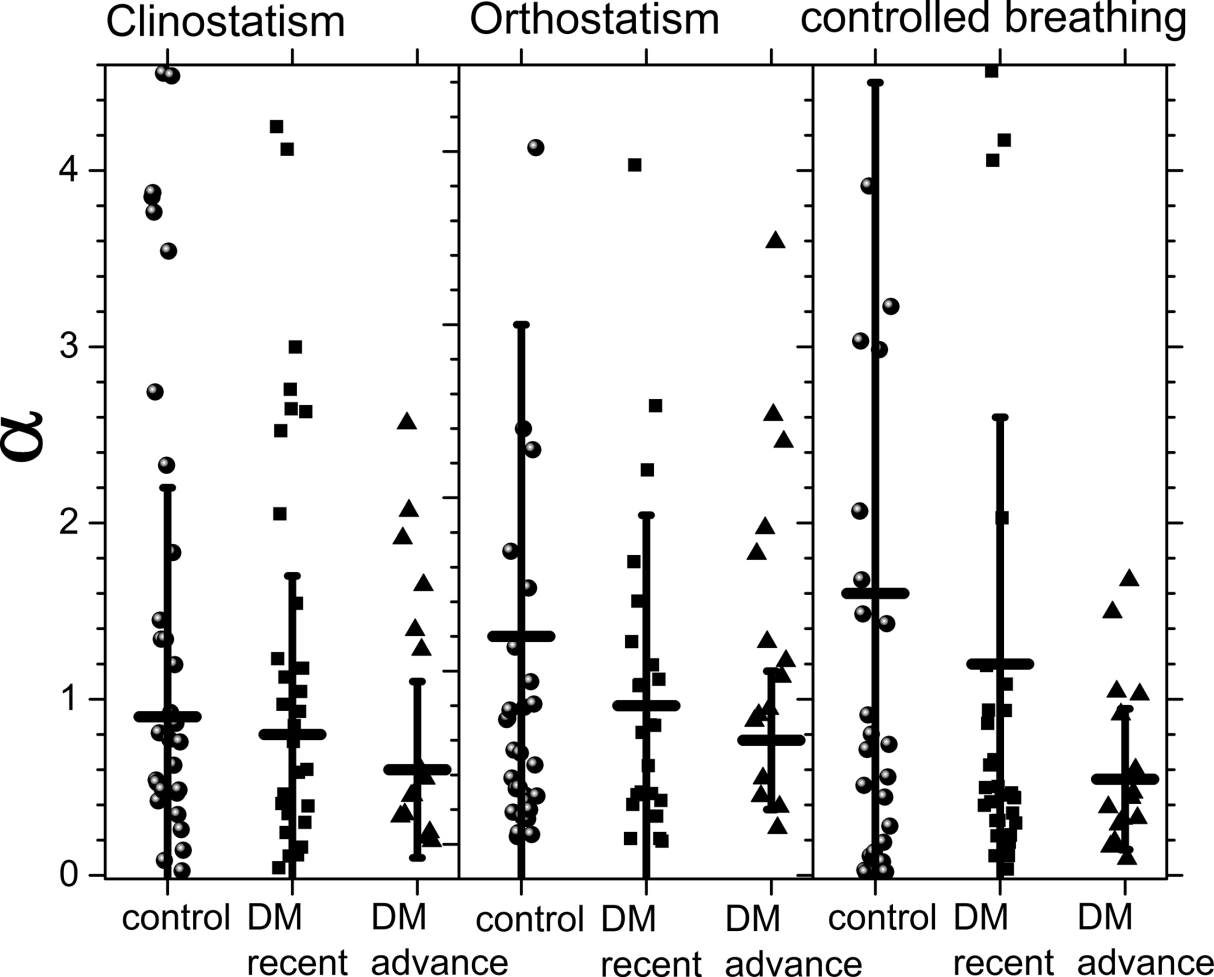
The α parameter for the different groups during clinostatism (left-hand panel), orthostatism (middle panel), and controlled breathing (right-hand panel). Crosshairs correspond to one standard deviation around the population average.

Fig 9 shows population averages of the parameter α and its standard deviation during all tests, while its values are given in Table 3. Controlled breathing allows to distinguish the 3 populations. During this test, healthy subjects have higher values of α with large SD reflecting the correlation between IBI and SBP. As DM evolves, α diminishes not only in value but also in variability. That is, not only the heart response becomes more rigid, but the variability of the blood pressure also increases, in agreement with HRV being a protective health factor and blood pressure variability a risk factor. Long-standing DM patients have very similar values of α under all the tests, reflecting that for them is more difficult to respond to external changes, they have parameters that are more rigid. Using the t-student’s test, the hypothesis of distinct population averages of α for control subjects and DM patients is verified in the case of controlled breathing (see Table 3 and Fig 9). On the other hand, t-student’s test does not show a statistical significant difference for α between recently diagnosed and long-standing DM patients. This implies that our proposed α parameter can help to distinguish health from diabetes illness during a controlled breathing test.

**Table 3:** α parameter.

	Clinostatism	Orthostatism	Controlled breathing
Control	0.9 ± 1.3	1.2 ± 1.8	1.6 ± 2.9
Recently diagnosed DM II	0.8 ± 0.9	0.8 ± 1.1	1.2 ± 1.4
Long-standing DM II	0.6 ± 0.5	0.6 ± 0.4	0.6 ± 0.4

## Discussion

As DM evolves, a more rigid cardiovascular response is produced due to a loss of variability in the IBI signals for clinostatism, orthostatism and controlled breathing. This loss is manifested not only by a diminished variance, but also by a leptokurtic distribution. The leptokurtic distribution is due to the fact that as DM evolves, the IBI become shorter and do not change with positional changes or breathing. Finally, there is very little variability left, which may be due to the heart’s default function or to hemodynamic changes induced by the respiration.

During clinostatism, SBP variances of recently diagnosed DM patients present large fluctuations with large variances, and high asymmetry. SBP variability manifests itself as a platykurtic distribution for all the tests. Interestingly, SBP variability after long-standing DM returns to the characteristic values of the healthy subjects, with small fluctuations (SBP is a very stable physiological signal). It is important to note that long-standing DM patients usually take medications that regulate their glucose levels and blood pressure, and this could also help to adjust the fluctuations of their blood pressure as our measures of SBP variability show. In concordance with the physiological studies of Guyton and his group [43, 44] in dogs with sino-aortic denervation and baroreceptor’s damage, where the vagal-sympathetic baroreceptor of the SBP answer is retarded, a platykurtic SBP distribution results. We observed a similar effect as DM evolved. The loss of balance between vagal and sympathetic effects on the baroreceptor is also reflected in the variation of the skewness with respect to the median.

Moreover, the loss of balance between vagal and sympathetic influence on HRV also alters the high protective factor HRV and the low risk factor of the blood pressure variability in DM patients as reflected by the statistically significant difference between the α parameter for healthy subjects and DM patients under clinostatism. As DM evolves, the variability of the α parameter decreases reflecting that the system is more rigid. Thus, the α parameter seems to make a statistically significant distinction between healthy and DM individuals under clinostatism test. During this test, control subjects have higher values of α with large SD, indicating large intrapopulation variability. As DM evolves, α diminishes not only in value but also in variability, that is, the population becomes more homogeneous. The α parameter indicates that not only the heart response becomes more rigid, but also the variability of the blood pressure increases, in agreement with HRV being a protective health factor and blood pressure variability a risk factor.

## Conclusions

The analysis of IBI and SBP detrended time series in the time domain shows that all moments of the distribution are relevant parameters that allow significant differentiation between control subjects, recently diagnosed, and long-standing DM patients. In this paper it is shown that in the case of IBI, skewness and kurtosis with respect to the median are as effective parameters as the standard deviation in order to establish statistically significant differences between groups. As DM evolves, skewness with respect to the median tends to zero (increased symmetry), standard deviation decreases (higher rigidity), kurtosis also vanishes (data becomes more concentrated around the mean), and the distribution becomes more Gaussian. It is also important to notice that the dysfunction produced by DM gradually alters the relation between IBI and SBP response as shown by the decrease on α (defined here as the ratio between radius of IBI and SBP’s moments). The proposed α parameter can help to distinguish health from diabetes illness using a controlled breathing test. Moreover, for long-standing DM patients α has the same value under all tests reflecting the difficulty that these patients have in adapting to different stimulus. The data of this study shows that long-standing DM patients have considerable autonomic dysfunction. The heart rate variability data suggests that autonomic modulation of the heart is affected in long-standing diabetes, as shown by the reduction in standard deviation (increase in rigidity), skewness with respect to the median (symmetry measure, conventionally associated with the loss of the cardiac parasympathetic–sympathetic balance), and kurtosis (change to more leptokurtic distributions). Given that a reduction in heart rate variability indicators has been linked to an increased all-cause and cardiac mortality, it is quite likely that patients with diabetes who have a Gaussian HRV have deteriorated from an autonomic viewpoint and indicates that the autonomic neuropathy is progressive. These patients are probably at an increased risk of mortality. The analysis in this study is relatively simple to perform in patients, since it only employs the moments of the distributions and their ratios, so it can easily be adapted to clinical inspection.
